# Development of a Web-Based Intervention Course to Promote Academic Staff Well-Being: Protocol for a Mixed Methods Study Design

**DOI:** 10.2196/84729

**Published:** 2026-06-03

**Authors:** Henna Asikainen, Veera Lampinen, Nina Katajavuori

**Affiliations:** 1 Faculty of Education, University of Helsinki, Siltavuorenpenger 3, Helsinki, 00014, Finland, 358 503111941

**Keywords:** work-related well-being, psychological flexibility, acceptance and commitment therapy, online intervention, academic staff

## Abstract

**Background:**

Burnout and poor well-being are serious problems in the workforce all over the world. Especially in academia, high pressure is evident. Therefore, addressing burnout is essential for enhancing academic staff well-being. Acceptance and commitment therapy (ACT), which aims to enhance psychological flexibility skills comprising present moment awareness, value-based actions, and differentiation of thoughts from self, has been found to be a promising approach to enhance workers well-being and work satisfaction.

**Objective:**

The purpose of this study was to first develop and describe our acceptance and commitment therapy–based WELLS course, aimed at supporting academic staff well-being and working life skills, offered to academic staff. The second aim was to report the mixed methods study design protocol to explore the participants’ experiences of the effects of the course.

**Methods:**

We will conduct a single-arm, nonrandomized, and mixed methods study with baseline, postintervention, and 6-month follow-up assessments. We will use an explanatory sequential design in which qualitative data are used to explain and deepen the quantitative findings. The quantitative part of the study aims to recruit 300 participants from the University of Helsinki. Questionnaire data will be collected from the participants 3 times: at the beginning, at the end, and 6 months after the course for well-being–related measures such as burnout and working life skills such as time management and psychological flexibility. The participants will be clustered based on their initial burnout risk score using latent profile analysis. Differences in participants’ dropout, participation forms, and changes in well-being and working life skills during the course will be analyzed with mixed ANOVA. Qualitative data will be collected from reflective journals written at the end of the course, participants’ written goal statements, and interviews among willing participants. Reflective journals will be analyzed with qualitative content analysis, and interviews will be analyzed using thematic analysis.

**Results:**

As of March 2026, a total of 265 participants had enrolled in the course and given their consent to participate in the research. The current data are being analyzed, and the results are expected to be published in late 2026 or early 2027.

**Conclusions:**

If the intervention proves successful, it may serve as an important tool for improving academics’ well-being, working life skills, and engagement by understanding how this intervention can help academic staff members with different initial burnout levels. In addition, it can help to understand and solve individual challenges related to well-being in the academic workplace.

## Introduction

### Background

The work-related well-being of academics has been shown to be generally poor and has deteriorated over time [1]. Studies have reported, for example, that people working in education are at higher risk of work-related stress than those in other occupations [1,2]. There are several aspects influencing the well-being of academic staff, and they have experienced increased demands on their work because of, for example, an increasing number of students, technology-based learning, and diversity in the student population [3]. In addition, a decline in perceived job control has also been observed [1]. A recent review study by Shen and Slater [4] showed that the causes of academic staff stress are related, for example, to feelings of heavy workload, administrative problems, unfair rewarding, and program supervision. In addition, high expectations and a competitive atmosphere, including publication targets, were perceived as causing stress [4]. It is clear that academic staff are in need of services to support their well-being.

Well-being is a multilayered concept that is usually very difficult to define [5]. While a much-used concept, positive mental health is often used similarly with the term “mental well-being,” although they need to be considered separately [6]. The dual continual model of well-being considers not only the positive aspects of well-being but also the absence of negative symptoms in well-being [7]. The dual continual model of well-being divides well-being into hedonic and eudaimonic well-being, emphasizing both the absence of mental health problems and the presence of well-being [7]. The hedonic perspective on well-being emphasizes the subjective experience of positive emotions, such as pleasure and happiness, and the absence of negative emotions, as the eudaimonic perspective on well-being emphasizes the psychological functioning and living a meaningful life [8]. Mental well-being incorporates hedonic aspects, such as experiencing positive emotions (ie, feeling good), and eudaimonic aspects related to optimal functioning (ie, functioning well) in one’s life [8-10].

Job-related or occupational burnout can be conceptualized as an aspect of eudaimonic well-being and is a critical problem all around the world in occupational contexts [11]. Job-related burnout comprises 3 dimensions: exhaustion, cynicism related to one’s job, and reduced professional efficacy [12]. One way to conceptualize job-related burnout is the job demands–resources model, which indicates that burnout is a product of an imbalance between job demands and resources [13]. As mentioned earlier, academic staff can experience high demands in their work, such as an increasing number of students, a decline in perceived job control, administrative problems, unfair rewards, and supervision of programs [1,3,4]. Thus, the model highlights the importance of personal resources in maintaining the balance between resources and demands [13].

On the other hand, psychological flexibility, understood as opening up to difficult thoughts and emotions and moving toward important values, can act as a central personal resource [14]. Psychological flexibility describes the ability to recognize and accept difficult thoughts and emotions (instead of avoidance) mindfully and engage in value-based actions [14,15]. Psychological flexibility is the key concept in acceptance and commitment therapy (ACT), which is a form of third-wave therapy and aims to develop psychological flexibility [15]. ACT is grounded in functional contextualism, which seeks to understand psychological events (such as thoughts, emotions, and behaviors) by examining their function within a specific context rather than isolating them as individual entities [16]. This approach emphasizes the relationship between an event and its context to determine its purpose or role, assuming that behavior is not inherently good or bad; its value depends on the context in which it occurs [17]. Additionally, relational frame theory underpins this approach by focusing on how we understand and use language and thoughts through learned relationships between concepts [18]. Relational frame theory views cognition and language as processes of relational learning, where the meaning of an event or thought is shaped by its relationship to other events or thoughts [18].

Psychological flexibility is based on six basic processes: acceptance, cognitive defusion, being present, self-as-context, values, and committed actions [19]. *Acceptance* means willingly embracing thoughts and emotions as they occur without trying to change or avoid them, and *cognitive defusion* can be defined as seeing thoughts and emotions as mental events instead of truths [15]. *Being present* means engaging with the present moment instead of worrying about the past or the future, and *self-as-context* can be defined through viewing oneself as an observer of thoughts instead of letting them define oneself [19]. *Values* are meaningful aspects of our lives which can be identified and clarified, and *committed actions* mean taking purposeful actions in line with the values instead of going through life without thinking about what is important [14,15,19]. By helping individuals clarify their values and take purposeful actions, ACT fosters a sense of purpose and direction, which is associated with greater life satisfaction and well-being [15].

Over 40 years of research have demonstrated that psychological flexibility is associated with a range of positive outcomes, including improved mental health, enhanced emotional well-being, and greater life satisfaction [19]. It has been linked to reduced symptoms of depression, burnout, and anxiety, increased quality of life, and better coping strategies in the face of workplace stress [20-22]. The application of ACT in workplace settings has gained considerable attention, as organizations increasingly recognize the importance of employee well-being for both productivity and job satisfaction [23]. Studies have shown that ACT interventions can effectively reduce workplace stress, improve mental health outcomes, and enhance overall well-being [20,24]. For instance, Flaxman and Bond [25] conducted a randomized controlled trial examining the impact of an ACT-based stress management program on public sector employees. The results revealed significant reductions in stress and anxiety, as well as improvements in job performance and psychological flexibility [25]. Participants reported greater acceptance of challenging work-related thoughts and feelings, which facilitated more adaptive coping strategies [25]. Similarly, a meta-analysis by A-Tjak et al [26] highlighted the efficacy of ACT in occupational settings, demonstrating moderate-to-large effect sizes for improvements in psychological flexibility and reductions in work-related stress and burnout. The analysis underscored the potential of ACT to foster a supportive work environment that prioritizes employee well-being [26]. Although derived from nonacademic work contexts, these findings suggest that ACT may also be a valuable intervention for academic workers.

Despite the positive effects of ACT-based interventions on workers’ well-being, there has been a lack of such interventions targeted at academic staff, especially in online intervention formats [21,27]. Online interventions allow individuals to engage in more flexible support through materials that are constantly available, which may help to address barriers to seeking help, a key challenge for many who could benefit from such interventions [28]. Moreover, online interventions may be particularly valuable in workplaces with a large or geographically dispersed workforces, also including those with remote or hybrid work models (such as universities), as online programs also require fewer physical resources [29]. Thus, online interventions may provide substantial potential to be more scalable, accessible, and cost-effective than in-person interventions [28-30].

Although used more rarely, online ACT-based interventions have already shown promise in effectively improving mental well-being in a variety of contexts [28], including occupational contexts [29]. Despite this, far fewer online ACT-based interventions have been offered to workers [21], and to our knowledge, no current publications explore online ACT-based interventions for university staff specifically, regardless of their potential.

Although increasingly accessible, a common issue among online intervention research is participant engagement [21]. Engagement in this context refers to how users interact with an online intervention, namely through participants’ adherence to the intervention, particularly within the context of a study [31]. Specifically, this indicates initial uptake via enrollment and early interaction with intervention features, and the extent to which participants continue to use the intervention as intended over time. Low engagement and dropping out of the intervention before the intended end of the intervention often disrupt the ability of online programs to deliver effective treatment and complicate research conclusions [27,28].

Despite this, factors such as baseline symptoms of, for instance, burnout and whether the online intervention is completely self-guided (indicating no live interaction with others) or partially guided (indicating a form of live interaction during the intervention through a facilitator or peer interaction) have been found to potentially support engagement in online interventions [21,27]. Thus, exploring individual differences in participants’ experiences, such as baseline burnout, and the format of delivery may help clarify what types of online interventions work, and for whom, in terms of adherence and dropout within a study context.

In addition to psychological flexibility, effective time management is crucial for academics to balance their multifaceted responsibilities and maintain well-being, and thus should be considered when designing interventions for academic staff. Time management involves setting goals, prioritizing tasks, and allocating time efficiently [32]. A recent meta-analysis [33] showed that time management was moderately related to job performance, academic achievement, and well-being, and that time management also showed a negative relationship with distress. According to this study, time management enhanced well-being to a greater extent than performance. Another meta-analytic review showed that time management was associated with a variety of beneficial employee outcomes, such as increased job satisfaction, job performance, and lower levels of stress and burnout [34]. Thus, managing time can directly improve one’s work-life balance. Time management training can enhance academics’ ability to prioritize tasks and manage workloads effectively [35].

When developing online interventions, pedagogical aspects are also important to consider. Reflective learning and experiential learning are 2 pedagogical approaches, which both emphasize the importance of personal experience and reflection in the learning process and in promoting deeper understanding [36,37]. Schön [36] introduced the concept of the “reflective practitioner,” highlighting the importance of reflection-in-action and reflection-on-action as mechanisms for professional growth and development, whereas Kolb’s [37] model underscores the interplay between experience and reflection, suggesting that effective learning requires individuals to engage in reflective observation to derive meaning from their experiences.

### Aims and Objectives

There is a need to enhance academics’ well-being, yet effective, research-based, and feasible tools are limited. Accordingly, this study pursues 4 goals. First, we aim to develop an online ACT-based intervention for academic staff that integrates working-life skills, specifically time management and reflective skills, and is feasible to complete within demanding work environments. Second, we aim to evaluate the intervention quantitatively by identifying latent burnout profiles among university staff and examining whether changes in burnout, psychological flexibility, perceived stress, overall well-being, and procrastination differ across profiles and participation formats. Third, we aim to examine participant engagement and retention by investigating whether dropout differs across latent burnout profiles and according to participation formats (individual vs group-based). Fourth, we aim to explore participants’ lived experiences of the intervention qualitatively, with a particular focus on psychological flexibility processes and on which of these processes’ participants perceive as most meaningful for their well-being and functioning at work.

Our research questions (RQs) are as follows:

- RQ 1. What latent burnout profiles can be identified among staff at the University of Helsinki, and to what extent do the effects of the online ACT-based intervention differ between them?
- RQ 2. How do observed pre-post changes in burnout, psychological flexibility, perceived stress, and overall well-being vary across burnout profiles and participation formats?
- RQ 3. How do latent burnout profiles differ in training dropout rates, and does participation in group-based versus self-guided online ACT-based training relate to dropout?
- RQ 4. How do participants describe their experiences of psychological flexibility processes following participation in a digital ACT-based intervention, and which of these processes do they perceive as most meaningful in supporting their well-being and functioning in their work?

## Methods

### Study Design

#### Mixed Methods Design

We will conduct a single-arm, nonrandomized, and mixed methods study with baseline, post-intervention, and 6-month follow-up assessments. Our study methodology description is based on the GRAMMS (Good Reporting of a Mixed Methods Study) reporting guidelines [38] and checklist (Checklist 1). A mixed methods approach is chosen, as the study aims to evaluate changes in participants’ well-being and related outcomes at the group level and to understand how participants experienced the intervention and its psychological flexibility processes at the individual level. An explanatory sequential design will be used, with priority given to the quantitative strand. Quantitative data will address latent burnout profiles (RQ 1), changes in outcomes, and dropout (RQs 2 and 3), whereas qualitative data will be used to explain, contextualize, and deepen these findings by examining participants’ final reflections, goal statements, and interview accounts (RQ 4). Although some qualitative material is generated during the course, qualitative interviews will be conducted after the main quantitative data collection phase.

Integration will occur at the interpretation stage once all data have been collected. Integration will be conducted by the researchers, involving the comparison and combination of quantitative findings (RQs 1‐3, changes in outcomes, burnout profiles, and dropout) with qualitative data (RQ 4, participant reflections and interviews). Through this, we will explore how quantitative patterns are potentially explained and contextualized by participants’ individual experiences, as well as identify possible convergence, complementarity, or divergence between the 2 data strands.

#### Setting and Participants

This study is conducted at the University of Helsinki where the course is open to all university personnel, including teaching faculty, researchers, and administrative and support staff. The course has been available since fall 2023. For RQs 1 to 3, participants are recruited to attend the course via regular communications and advertisements to university personnel via university HR, intranet, email, and website. All willing participants can attend the course, and there are no exclusion criteria in the study. Participation in the research is optional, and participants can study in the course without consenting to research. At the beginning of the course, participants are asked to give or decline their permission to use their questionnaire data and written responses for research purposes.

For RQ 4, a purposive subsample (N≈30‐40) will be recruited from participants who have completed the course for semistructured interviews. Sampling will be stratified according to the latent burnout profiles identified in RQ 1 in order to include participants from each profile and permit systematic comparison of experiential patterns across profiles. Separate written consent is given for the interviews before the start of the interview.

For RQs 1 to 3, data collection started in 2023 when the course was opened to all staff members. For RQ 4, data collection is scheduled to take place in fall 2026.

### The Course

The course “Occupational Well-being for University Staff” is based on ACT, with a central aim of enhancing participants’ skills in psychological flexibility. Training in key working life skills, such as time and effort management and reflection, is integrated into the course. The course was developed on the web-based Moodle platform and can be completed entirely online. Although it is recommended to complete the course over a 12-week period, participants are free to proceed at their own pace. The flexible schedule is an attempt to accommodate the varying time demands of academic work and allow participants to engage with the course at times that best suit their individual schedules. Participants can take the course either with a group or on their own. A group can be, for example, a research group, a team, or a unit. The small group discussions in the course work best if there are 3 to 5 people in the group, but participants can choose how to conduct this. The course is designed to function without a facilitator.

### Course Modules

The course consists of 6 modules. Each module includes all necessary materials, such as instructions, short video lectures, practical exercises, reflective assignments, and guidance for peer discussions. The course is structured so that participants complete 1 module at a time.

#### Introductory Part

In the beginning of the course, the participants are introduced to the working in the course and informed about the research concerning the course. The research permit is also asked in the beginning. In addition, the participants are asked to answer questionnaires concerning their well-being and working skills, from which they receive feedback.

#### Module 1: Time Management

This module includes an introduction to the concept of psychological flexibility and a time management exercise in which participants are instructed to track their time use for a week. This exercise and the insights gained from it serve as a foundation for several reflective assignments throughout the course.

#### Module 2: Values

This module consists of materials and exercises that are related to one’s values and includes exercises that help participants to explore their values and think about the issues that are important for them. Participants can choose from a variety of exercises the ones that best suit them, and all exercises have been designed to help participants to recognize the issues and things that are important for them and to reflect on different areas of their lives and their current level of satisfaction with each. Based on this reflection, they are asked to formulate a personal goal for the course.

#### Module 3: Recognizing Thoughts

This module is focused on identifying one’s thoughts and understanding their effects on behavior. Procrastination, its influence on behavior, and strategies to overcome it are also discussed. Participants can choose from a variety of exercises tailored to their needs.

#### Module 4: Focusing on the Present Moment

This module emphasizes the importance of being able to concentrate on the present and develop awareness of one’s current feelings and emotions. Participants are encouraged to confront all varieties of their emotions, including negative ones, with acceptance and curiosity. Additionally, stress, its effects, and strategies to overcome it are discussed in this module.

#### Module 5: Self-Compassion

This module focuses on self-compassion, comprising themes related to self-kindness, acceptance, and the self as context. Participants are also encouraged to cultivate a thankful attitude in their daily lives and practice compassion toward their peers.

#### Module 6: Committed Action

**Figure 1.**
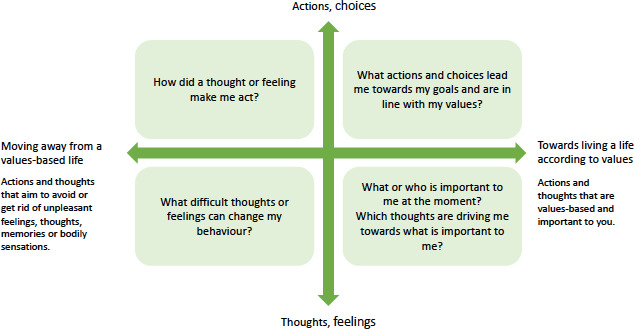
Square field for value-based actions.

The last module is related to committed actions and addresses how one’s values can be concretely translated into action. As a weekly assignment, the participants are asked to reflect on value-based actions in their lives according to the square field below (Figure 1)—thinking about their actions, emotions, and thoughts toward meaningful actions. Participants are instructed to consider how they will move forward after the course and what new goals they might set for themselves. In addition, the participants assess their well-being and working skills again and are encouraged to reflect on their change in the scores and overall development during the course.

### Measures and Data Collection

#### Quantitative Data

First, for RQs 1 to 3, validated self-assessment questionnaire data will be collected before (T1), after (T2), and 6 months after the course (T3). The questionnaires measure emotional, psychological, and social well-being (Mental Health Continuum-Short Form) [39]; psychological flexibility (Work-related Acceptance and Action Questionnaire) [40]; perceived stress (Perceived Stress Scale) [41]; and risk of burnout (15-item Bergen Burnout Indicator) [42]. Psychometric properties will be further evaluated in the present sample via internal consistency (Cronbach α) and factor structure (confirmatory factor analysis). A 6-month follow-up is used to examine how potential changes during the course persist or change over time. Psychological flexibility especially is understood as a process that develops gradually through ongoing practice and integration into daily life, and changes may therefore continue to evolve beyond the course period [21]. Furthermore, this timeframe is also consistent with prior research in the field, which commonly uses follow-up periods of 6 months [43-45].

#### Qualitative Data

Subsequently, for RQ 4, the study will use qualitative data to deepen insights derived from quantitative data. Data will be collected from (1) participants’ written final reflections submitted on the course platform, (2) participants’ written goal statements completed at the beginning and end of the course, and (3) semistructured interviews conducted after the end of the course.

In the beginning, the participants are asked to analyze and identify what factors cause them stress in the workplace or in life and how they could affect it. They are also asked to set a value-based goal which they focus on during the course. At the end of the course, participants write a final individual reflection on how they experienced the course, how the course affected their well-being and working life, how they achieved their goal, and how they are planning to go forward.

Semistructured interviews will examine participants’ experiences of psychological flexibility processes following participation in the intervention, with particular attention to which processes are perceived as most meaningful in supporting well-being and functioning in academic work. The interview guide will be informed by the ACT model and will address core psychological flexibility processes, including acceptance, cognitive defusion, present-moment awareness, self-as-context, values clarification, and committed action, while allowing participants to elaborate on how these processes were experienced and applied in their work context.

### Analyses

#### Statistical Analyses

For RQ 2, the effects of the course are analyzed with repeated-measures ANOVA and hierarchical modeling to see the changes at the group level. In addition, for RQ 1, the participants will be clustered based on their initial burnout risk score to compare the effects of the course in different risk groups using latent profile analysis and repeated-measures ANOVA. For RQ 3, attrition analyses are conducted using chi-square tests of independence and odds ratios to examine whether dropout rates differ across latent burnout profiles and between group-based and self-guided participation formats.

#### Qualitative Analysis

For RQ 4, the final reflections will be analyzed and categorized with inductive content analysis to explore the different reported effects that the course had. In addition, the participants’ goals will be analyzed at the beginning and the end of the course, examining how the goals were achieved and what factors influenced changes in behavior. These will also be used to analyze the benefits of each module on different participants. These data will be combined to get a deeper understanding of the effects of the course and the changes in the participants’ behavior.

Interview data will be analyzed using reflexive thematic analysis following Braun and Clarke [46]. The qualitative data will also be analyzed using reflexive thematic analysis following Braun and Clarke [38,46]. The analysis will adopt a primarily data-driven (inductive) approach, whereby patterns and shared meanings are identified from participants’ accounts rather than imposed a priori.

The analytic process will proceed in several phases. First, the researcher will engage in repeated reading of the interview transcripts to achieve familiarity with the data, while documenting preliminary observations and analytic reflections. Second, systematic coding will be conducted across the entire dataset. Segments of text relevant to the research questions will be identified and color-coded, with codes representing meaningful units of data that capture aspects pertinent to participants’ experiences of burnout and psychological flexibility processes. Following initial coding, codes from all interviews will be collated and examined comparatively. Through iterative review and refinement, potential themes will be developed to represent shared patterns of meaning across participants. Theme development will focus on conceptual coherence with the theoretical framework of psychological flexibility and relevance to the research questions rather than frequency alone, consistent with Braun and Clarke’s [38] guidelines. Themes will be reviewed, defined, and refined to ensure internal consistency and clear distinction between themes, resulting in a structured thematic representation of participants’ lived experiences.

### Ethical Considerations

A statement of ethical acceptability has been given to the research plan of the course by the University of Helsinki’s Research Ethics Committee in the Humanities and Social and Behavioral Sciences (statement 75/2024), and the research will be conducted according to the Finnish National Board on Research Integrity (TENK) guidelines [47]. Informed consent from all the participants is obtained at the beginning of the course, which is signed online. The participants are informed about the study with a study information sheet in the course area. In addition, a data privacy notice is given to the participants in the course area detailing the research protocol, data collection and management, and participants’ rights following the General Data Protection Regulation of the European Union. Participants are informed about the possibility to withdraw from the research at any time, and the course can be completed without participation in the research.

For RQs 1 to 3, all data are collected in the secured course area and downloaded and backed up on secure servers with restricted and password-protected access. The data comprising the questionnaire data used in the study are pseudonymized before analysis. Participants are distinguished in the data by an identification number. The identifiers are not shared outside the research group. The contact information of the participants is stored separately from the research data. The qualitative interview data are recorded and transcribed. The transcriptions and the written assignments used in research will be anonymized as much as possible by removing locations and other identifying information from the transcripts; names are replaced with identification numbers. The data with identifiers will be destroyed 10 years after the end of the research project.

For RQ 4, all interview data will be collected via the University of Helsinki’s Zoom, where interview sessions are recorded. The data will be pseudonymized during transcription, and any potentially identifying details (such as names, colleagues, units, or departments) will be removed. The data is backed up on secure servers with restricted and password-protected access. Only the research team will have access to the recordings and transcripts. Audio recordings will be deleted after transcription. Neither the employer nor any other third party will have access to interview material, and no individual participant will be identifiable in any reports, publications, or presentations. We will emphasize to participants that their employer will not be informed of their participation or responses, and participation or nonparticipation will have no impact on their employment.

## Results

As of the first data freeze in November 2025, a total of 265 participants had enrolled in the course and given their consent to participate in the research, and 113 participants have completed the course (Table 1). Among participants who completed the course, the mean number of days between questionnaires at T1 and T2 was 123, and the median was 109 days. The first data cut is expected to be published in late 2026 or early 2027. Preliminary results suggest that participants’ burnout scores at T1 reflect 3 distinct profiles and that the profiles experiencing the highest burnout scores are also more likely to drop out. Similarly, participants completing the course alone were more likely to drop out than those in a peer group.

**Table 1.** Characteristics of the study sample recruited in the first data cut (N=265).

Background characteristics	Participants, n (%)
Job description	
Teaching and research staff	35 (13.2)
Other expert and support staff	226 (85.2)
Missing or N/A^a^	4 (1.5)
Course completion method	
Self-guided or alone	99 (37.4)
With a peer group	145 (54.7)
Missing or N/A	21 (7.9)

a N/A: not available.

## Discussion

### Overview

The aim of this paper was to develop an ACT-based well-being and working life skills course for academic staff, as well as to present our study design. The course was developed based on research on how to support academics’ well-being and working life skills to answer the current well-being problems they are facing [3]. Thus, the course was based on the principles of ACT [15], further fostering the development of psychological flexibility and working life skills.

During this course, each subprocess of psychological flexibility is addressed across the modules. In addition, several working life skills–related themes are interwoven throughout the entire course. We have built the course with pedagogical tools to support participants’ learning and engagement, in particular guided reflection and collaborative peer group work.

### Principal Findings

We anticipate that participants’ baseline burnout levels will be heterogeneous and will reflect at least 2 distinct burnout profiles. We also hypothesize that academic staff will report improvements in their well-being at the end of the course and that dropout rates are significantly different between different burnout profiles and participation formats.

### Comparison to Prior Work

ACT has been studied widely among university students [48-51] and increasingly in wider occupational contexts [21,22]. However, to our knowledge, there is very limited research examining ACT-based online courses for university staff specifically.

### Strengths and Limitations

This study’s key strength lies in its versatile mixed methods approach. First, by using latent profile analysis to profile participants’ baseline burnout scores (RQ 1), we explore burnout as a complex and heterogeneous construct and move beyond aggregated group-level averages to a more person-oriented perspective, which has gained more popularity in the last decades [52]. We aim to additionally explore participants’ self-reported changes during the course quantitatively (RQ 2) but also deepen and contextualize these findings with qualitative insights (RQ 4). This integration allows us to identify not only whether change occurs but also to explore how and why participants perceive change happens or does not happen, and whether qualitative experiences converge with or diverge from quantitative trends. Thus, the study may provide a more nuanced understanding of what participants experience during the course and which aspects may be most useful for them. Finally, we also aim to examine whether burnout profiles and the format of participation may influence engagement in the course (RQ 3), which may provide practical information regarding how and to whom the course may be most beneficial.

Furthermore, the study design also benefits from its flexibility, as well as its ease of execution and implementation in university work environments. As the study takes place in an online environment with no facilitation, a flexible schedule, and is continuously open, the study does not require excessive resources and is relatively easy to carry out. Additionally, the flexible design may mitigate some of the burden on participants, as they are able to complete the course at their own pace, thus also following ethical research principles.

However, the study design also possesses limitations. The flexible schedule design also indicates that a control group cannot be used, and thus we are not able to randomize participants, which precludes, for instance, a randomized controlled trial. A nonrandomized design may also introduce selection bias, which may limit the generalizability of the results. Therefore, the conclusions regarding the effectiveness of the course are limited.

A potential limitation of the mixed methods design is that qualitative data are collected from a subsample, which may not fully represent the broader quantitative sample, potentially limiting the integration of the findings and the generalizability of the results.

We additionally recognize that participation in the course may introduce extra time demands for already busy academic staff, particularly given the inclusion of assignments, reflections, questionnaires, and (optional) group discussions. Lack of time is a common issue affecting engagement in online interventions [21,27] and is also a relevant concern in this study. Thus, although the course is designed with flexibility in mind, with participants being able to progress at their own pace, the time commitment needed to complete the course may still lead to excessive dropout. However, the study design attempts to address this by considering dropout, specifically by exploring whether additional factors, such as baseline burnout scores and the format of participation (self-guided vs. with a peer group), may contribute to this.

### Future Directions

Future research could address some of these limitations by investigating an iteration of the course that provides more structure via, for instance, facilitated sessions organized by the researchers, a predetermined time window for participation, and a control group for more robust investigations of effectiveness. Moreover, future research should more explicitly assess the perceived time burden of the course and how intervention components and workload can be best aligned with the realities of academic work.

Furthermore, recent process-based approaches to ACT underscore that changes in well-being unfold over time within individuals rather than between time points and group averages and should thus be examined beyond pre-post measures [53]. As psychological flexibility is assessed only at the beginning and end of the course, this study does not idiomatically examine how change occurs during the intervention, especially for the individual. Future studies could therefore incorporate daily, within-person process-based assessments (such as the Process-based Assessment Tool [54]) to more comprehensively capture how these processes may vary individually over time.

### Dissemination

The results from this research will be published in scientific peer-reviewed journals as well as scientific conferences.

## Supplementary material

10.2196/84729 Checklist 1 GRAMMS checklist.

## Abbreviations

ACT: acceptance and commitment therapy
GRAMMS: Good Reporting of a Mixed Methods Study
RQ: research question
