# Supplementary material for: Development of a Web-Based Intervention Course to Promote Academic Staff Well-Being: Protocol for a Mixed Methods Study Design
Source: JMIR Res Protoc. 2026 Jun 3;15:e84729. doi: 10.2196/84729 (PMC13232920; doi:10.2196/84729)
Supplement: Checklist 1 [file resprot-v15-e84729-s001.docx]

**Appendix A. Good Reporting of A Mixed Methods Study (GRAMMS) checklist**

| **Guideline** | **Section: page** |
| --- | --- |
| Describe the justification for using a mixed methods approach to the research question | Methods; Mixed-methods design: p. 9-10 |
| Describe the design in terms of the purpose, priority and sequence of methods | Methods; Mixed-methods design: p. 9-10 |
| Describe each method in terms of sampling, data collection and analysis | **Sampling;**  Methods; Setting and participants: p.10-11  **Data collection;**  Measures and data collection; Quantitative data: p. 14-15  Qualitative data: p.15  **Analyses;**  Statistical analyses: p. 16  Qualitative analyses: p. 16-17 |
| Describe where integration has occurred, how it has occurred and who has participated in it | Methods; Mixed-methods design: p. 9-10 |
| Describe any limitation of one method associated with the present of the other method | Discussion; Strengths and limitations: p.20-22 |
| Describe any insights gained from mixing or integrating methods | Discussion; Strengths and limitations: p.20-22 |
